# Identification of Rare Causal Variants in Sequence-Based Studies: Methods and Applications to *VPS13B*, a Gene Involved in Cohen Syndrome and Autism

**DOI:** 10.1371/journal.pgen.1004729

**Published:** 2014-12-11

**Authors:** Iuliana Ionita-Laza, Marinela Capanu, Silvia De Rubeis, Kenneth McCallum, Joseph D. Buxbaum

**Affiliations:** 1 Department of Biostatistics, Columbia University, New York, New York, United States of America; 2 Memorial Sloan-Kettering Cancer Center, New York, New York, United States of America; 3 Seaver Autism Center for Research and Treatment, Icahn School of Medicine at Mount Sinai, New York, New York, United States of America; 4 Departments of Psychiatry, Mount Sinai School of Medicine, New York, New York, United States of America; 5 Departments of Genetics and Genomic Sciences, and Neuroscience, and Friedman Brain Institute, Icahn School of Medicine at Mount Sinai, New York, New York, United States of America; 6 Mindich Child Health and Development Institute, Mount Sinai School of Medicine, New York, New York, United States of America; Georgia Institute of Technology, United States of America

## Abstract

Pinpointing the small number of causal variants among the abundant naturally occurring genetic variation is a difficult challenge, but a crucial one for understanding precise molecular mechanisms of disease and follow-up functional studies. We propose and investigate two complementary statistical approaches for identification of rare causal variants in sequencing studies: a backward elimination procedure based on groupwise association tests, and a hierarchical approach that can integrate sequencing data with diverse functional and evolutionary conservation annotations for individual variants. Using simulations, we show that incorporation of multiple bioinformatic predictors of deleteriousness, such as PolyPhen-2, SIFT and GERP++ scores, can improve the power to discover truly causal variants. As proof of principle, we apply the proposed methods to *VPS13B*, a gene mutated in the rare neurodevelopmental disorder called Cohen syndrome, and recently reported with recessive variants in autism. We identify a small set of promising candidates for causal variants, including two loss-of-function variants and a rare, homozygous probably-damaging variant that could contribute to autism risk.

## Author Summary

Sequencing technologies allow identification of genetic variants down to single base resolution for a whole human genome. The vast majority of these variants (over 90%) are rare, with population frequencies less than 1%. Furthermore, in a specific study, many of the variants identified are not associated with the disease of interest, and identification of the small proportion of truly causal variants is a difficult task. Clearly, for causal variants that are rare enough to only appear a few times in a study, observed frequencies in cases and controls are not enough to distinguish them from the vast majority of random variation, and rich functional annotations can help identify the causal variants. Here we propose to develop a set of statistical methods that leverage diverse functional genomics annotations with sequencing data to identify a small set of potentially causal variants and estimate their effects. Pinpointing a subset of potentially causal variants is crucial for understanding precise biological mechanisms, and for further experimental functional studies.

## Introduction

The tremendous progress in massively parallel sequencing (aka ‘next generation’ sequencing) technologies enables investigators to obtain genetic information down to single base resolution on a genome-wide scale in a rapid and cost efficient manner [1], [2], [3]. The resulting datasets are high dimensional and very sparse, with millions of genetic variants, the vast majority of which are rare in the population. For example, in any genetic region, it is expected that over 90% of genetic variants have a frequency in the population of less than 1% [4]. Therefore in any given study, most variants are only observed a small number of times (e.g. many of them are singletons or doubletons). This sparse nature of the data poses nontrivial statistical difficulties, and traditional statistical methods employed for association testing with common variants are not powerful in this context [5].

Both empirical and theoretical studies suggest that rare genetic variants are an important contributor to disease risk [6], [7], [8], [9]. Over the past few years several statistical tests have been proposed to test for association with rare variants in a small genetic region, such as a gene [10], [11], [12], [13], [14], [15], [16], [17]. The proposed association tests are based on the idea of grouping together variants in the gene, and testing for association at the gene rather than variant level. While these methods attempt to increase power by cumulating the signal across a larger region, they compromise precision and, in particular, it is not possible to pinpoint individual causal variants and estimate their effects on disease. Prioritizing a small number of plausible causal variant candidates is very important for further follow-up functional studies, since experimental analyses are difficult to implement and expensive for large number of variants [18], [19]. Furthermore, identification of causal variants is essential for understanding the precise molecular mechanisms of disease. Despite its importance, this problem is only now possible to address due to the increasing availability of large-scale sequencing data and the advances in computational methods for predicting the functional effects of genetic variation [19].

The fundamental challenge in pinpointing rare causal variants is that these variants are observed very infrequently in any given dataset and these sparse frequencies on their own are insufficient to provide meaningful risk predictions. In particular, for singletons or doubletons it will be necessary to incorporate prior functional and evolutionary conservation information in order to prioritize them as likely causal. Hierarchical modeling offers a natural strategy to leverage collective evidence from rare variants with sparse data. This can be accomplished in the presence of hierarchical covariates that are associated with disease risk and which can be used for implicitly aggregating the rare variants to permit stronger inferences about individual variants. These hierarchical covariates are characteristics of the variants themselves, such as the degree of conservation across species, the position in the gene, and other features that can be represented using bioinformatic measures. Indeed, many annotation tools (such as ANNOVAR [20], PolyPhen-2 [21], SIFT [22], GERP++ [23]) exist to predict the possible impact of a variant on the function of a human protein, or the level of evolutionary constraint for a variant. Even though such bioinformatic predictions of deleteriousness are not extremely accurate and are continuously being improved, they can provide useful information on the prior likelihood that a variant is causal, especially when multiple such predictors are used, as we show in this work. In earlier work we have developed a hierarchical modeling approach that is capable of estimating odds ratios for variants that occur infrequently in the dataset [24], [25]. Hierarchical regression techniques have also been adopted in a Bayesian framework with the goal of detecting rare causal variants [26], [27], however they can be computationally intensive and can be dependent on the choice of the prior weights [27]. More recently, Pickrell [28] has used hierarchical models to combine rich functional genomics annotations (as generated by the ENCODE project [29]) and summary statistics from GWAS to identify types of genomic elements enriched among disease susceptibility loci.

Here we propose and investigate the performance of two complementary statistical methods that are able to incorporate prior information on the putative function of individual variants in a gene in order to (1) identify a list of likely causal variants, and (2) estimate the effects of these variants on disease. The first approach is a backward elimination procedure based on groupwise association tests that leads to the identification of a small set of “interesting” variants in the gene, which are enriched in causal variants. The second approach complements the first by employing hierarchical models [24],[25] that can incorporate diverse functional and evolutionary conservation annotations, and in turn provides effect size estimates and confidence intervals for individual variants.

## Methods

First, we review the basics of groupwise association tests, and then we describe in detail the two complementary methods we propose for prioritizing variants for follow-up functional studies.

### Groupwise association tests for sequencing data

We assume that *n* subjects have been sequenced in a region of interest (e.g., a gene), that contains *m* variants. Let 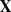 be the 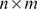 genotype matrix. We consider the regression model
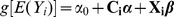
(1)where 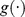 is a link function, and can be set to be the identity function when traits are continuous, or the logistic function when traits are dichotomous; 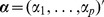 are regression coefficients for the covariates 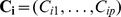 that we want to adjust for. 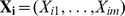 is the vector of genotypes for the *i*th individual, and 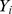 is its trait value. 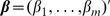 are regression coefficients for the *m* genetic variants.

We are interested in testing the null hypothesis of no genetic effects: 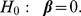 Testing each individual 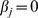 or using multiple df tests can lack power because of the sparsity of the data and the many variants in a gene. Therefore, we need to impose certain assumptions on 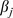
's to make the test more powerful. For example, one of the most widely used tests, the Burden test, assumes that all *β*'s have essentially the same value, say 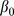, and the regression model in (1) amounts to 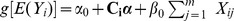. More generally, Lee et al. [15] assume that 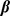 is a random variable with 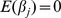, 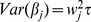 and 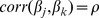 for different *j* and *k*. To test the null hypothesis of no genetic effects 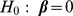 the variance-component score statistic has been proposed [15]:
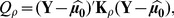
(2)where 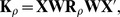 and 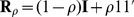 specifies an exchangeable correlation matrix, and 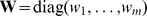 is a diagonal weight matrix, where each weight can be related, for example, to the predicted functional effect of a variant (e.g. PolyPhen-2 or SIFT score); for a dichotomous trait, 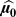 is a vector of estimated probabilities of 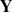 under the null model. Although this class of tests is more general, the two commonly used tests are the Burden test (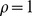) and the SKAT test (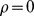). These score statistics are easy to compute and can be written simply as



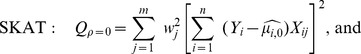
(3)

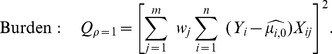
(4)


The null distribution of 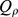 is approximated by a mixture of 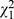 distributions. Davies' method [30] or moment matching can be employed to calculate the p value. The relative performance of the two tests will depend on the true underlying disease model. The Burden test tends to be more powerful when disease associated variants are all of the same type (risk or protective) and with effects of similar magnitude. The SKAT test tends to be more powerful when there is a mixture of risk and protective variants, and also when only a small percentage of variants in a region are causal. A parallel framework for family-based designs has also been proposed [16].

### Backward elimination procedure

The groupwise association tests described above test for association at a gene level, but are not able to pinpoint individual causal variants in the gene. However, once a gene has been shown to contain variants associated with disease (e.g. using the Burden or SKAT tests), identifying the individual causal variants among the many variants in a gene is of considerable interest as it can lead to a better understanding of the molecular mechanisms underlying a complex trait, and is essential for further experimental validation work.

Starting with a groupwise association test, one natural way to identify causal variants that are individually of weak effect is to evaluate their contribution to a given set of variants by removing the variant from the set, and assessing the resulting effect, e.g. the p value for the reduced set. The following iterative algorithm (essentially a backward elimination procedure) is designed for this purpose.

**Backward Elimination Algorithm:** Step 1. Start with a set of 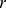 variants 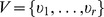. The current set is 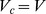. Compute the score statistic 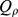 in eq. (2) (either 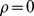 or 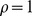) for this current set 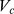, and compute the p value: 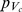.

Step 2. Remove each of the 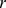 variants one at a time from 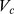, i.e. consider the sets 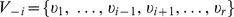 with 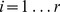, and then compute the corresponding score statistic and p value for each of these reduced sets: 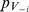.

Step 3. If 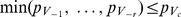 then remove the variant 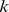 that leads to the smallest p value:
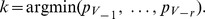



The current set becomes 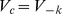 and repeat steps 2 & 3. If the current p value cannot be improved, then go to step 4.

Step 4. Return the current set of variants.

The results on a typical simulated example are shown in Figure S1. We show there the effect of removing a causal variant on the p value of the reduced set (i.e. 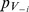 in Step 2 above), compared to removing a non-causal variant. As shown, the removal of causal variants will tend to result in an increase in the p value for the reduced set, as desired. There is a highly significant difference in the p values for the reduced sets when removing causal vs. non-causal variants (bootstrap Kolmogorov-Smirnov test p value 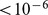).

This algorithm is applicable when the number of variants we start with in Step 1 is not too large (otherwise, the contribution of a weak variant to a large set is difficult to evaluate). However, sequencing a gene in thousands of individuals can lead to the detection of potentially hundreds of variants, or more. Therefore, we use a resampling procedure, whereby each time a small number of variants is chosen (say 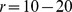) from the large number of variants identified in a gene, and then the above algorithm is applied to such small sets a large number of times (in our examples we use 2000 such re-samplings, although this number can be increased in the case of a large number of variants in the gene). At the end, for each variant in the gene we calculate the number of times it was returned in Step 4; we call this number the return count for a variant. A similar resampling procedure has been applied before in the context of gene-by-gene interaction [31]. Our goal is to use the sample of return counts to partition variants into two groups: “interesting” (higher return counts) and “non-interesting” (lower return counts), with the “interesting” category expected to be enriched in disease causing variants. We use nonparametric EM-like methods [32] to identify the two subgroups (see Text S2 for more details).

#### Integrating functional annotation into the above algorithm

It is well recognized that certain functional categories are more likely to be enriched among causal variants than others [28], [33]. An obvious example is rare non-synonymous variants, which are known to be enriched among disease causing variants. Similarly, loss-of-function (LoF) variants, including nonsense, splice-site and frameshift mutations, are heavily enriched among causal variants [18]. Therefore stratifying variants by different functional categories can improve false discovery rates. We can incorporate information on functional annotation in the backward elimination algorithm above. This can be done by simply applying the algorithm within different classes, say non-synonymous and synonymous. Furthermore, other functional or conservation scores (such as PolyPhen-2, SIFT and GERP++ scores) can be explicitly incorporated in the Burden and SKAT score statistics themselves (as weights associated with individual variants in eq. (2)), although only one such score can be incorporated at a time.

### Hierarchical model to estimate odds ratios of individual rare variants

A complementary approach to the backward elimination procedure described above is a hierarchical model. Hierarchical modeling has several important advantages in the analysis of rare variant data, because it can naturally integrate various functional prediction scores for individual variants. Such prior knowledge will be essential in pinpointing the likely causal variants in a gene, especially for causal variants that are rare enough to only appear a few times in a study (e.g. singletons and doubletons). For such variants, observed frequencies in cases and controls are clearly not enough to distinguish them from the vast majority of random variation (in the Nelson et al. study [4], more than 74% of variants were singletons or doubletons). Information on the putative functional effect of a variant on the protein or the degree of evolutionary conservation can be an important indicator on the likelihood of a variant being causal.

Such functional information can be incorporated through a hierarchical model [24], [25]. In the first stage, the trait value 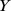 is related to the genotypes and possible confounders via the following model:
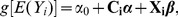
(5)with notations similar to those in model (1) above.

A second stage model relates the individual variant risks to prior (e.g. functional annotation) information known about the variants:
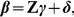
(6)where Z is an 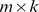 matrix for the *k* variant covariates (e.g. functional information); 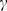 is a 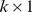 vector of regression parameters for the second stage covariates, and 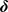 is a vector of normally distributed residual effects, assumed (for convenience) to be statistically independent. A principal advantage of the hierarchical modeling framework is that it can easily incorporate multiple functional annotations.

Combining the two models, one obtains the following generalized linear mixed effects model:
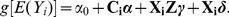
(7)


The parameters of this model can be estimated using a hybrid Bayesian pseudo-likelihood approach which performs Bayesian estimation of the variance component of the model and then conducts pseudo-likelihood estimation of the fixed and random effects using this estimated random effects variance [24], [25]. We can use the resulting estimates for the odds ratios and their standard errors to rank variants in a gene. Naturally the most difficult to identify are causal variants that occur only a few times. The odds ratio estimates for such variants will heavily depend on the higher level covariates, such as information on the predicted functional effect for a variant. For example, for a variant that occurs infrequently in a dataset (e.g. 2 times in cases and 0 times in controls), knowing that it is a LoF variant increases its likelihood to be a causal variant compared with a synonymous variant with the same frequency.

#### Backward elimination procedure and hierarchical model

We combine the two complementary methods, the backward elimination procedure and the hierarchical modeling framework, as follows. We identify the list of “interesting” variants from the backward elimination procedure, and for each of these variants we report the effect size estimate, and the associated standard error obtained from the hierarchical model (when applied to all variants, not just to the “interesting” list). The variants in the “interesting” list can be naturally ranked according to these effect estimates. We show below that restricting attention to only the list of “interesting” variants can improve the ranking of causal variants, and that this combined approach performs well in the scenarios we investigated.

Both methods are computationally efficient and have been implemented in software available on the authors' website (http://www.columbia.edu/~ii2135/).

## Results

We evaluate the performance of the proposed methods using simulated data and then apply them to two sequencing studies, the Dallas Heart Study and a study on Autism Spectrum Disorders.

### Simulated data

We simulated sequence data on 10,000 haplotypes in one genomic region of length 1 Mb under a coalescent model using the software package COSI [34]. The model used in the simulations was the calibrated model for the European population. For our purposes, we randomly sampled small subregions of size 10 kb, and simulated datasets with 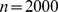 individuals (equal number of cases and controls). The number of variants and the minor allele frequency (MAF) distribution varies depending on the subregion sampled.

We considered two disease models (Table 1). In these two models, the odds ratio (OR) is a decreasing function of the MAF. For both models, we assume that 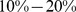 of the variants with MAF ≤0.05 in the 10 kb region under investigation are causal variants.

**Table 1:** Two disease models M1 and M2.

Model	Description
M1	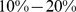 of variants with MAF 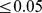 have OR 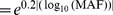
M2	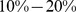 of variants with MAF 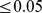 have OR 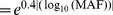

The odds ratio (OR) is a decreasing function of the minor allele frequency (MAF) at the causal variants.

For a dichotomous trait, we assumed the logistic model:
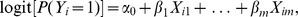
with 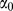 chosen such that the disease prevalence was 0.05.

We have also simulated bioinformatic covariates for variants to be used in the backward elimination algorithm, as well as to be incorporated in the hierarchical model. A first bioinformatic covariate we simulate is a binary variable, such as whether a variant is non-synonymous or not. Based on empirical studies [4], we consider the non-synonymous to synonymous ratio (NS:S) for the rare variants in the region to be between 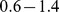 (depending on the strength of the purifying selection in the region). We assume that 80% of causal variants are non-synonymous, and then using Bayes' rule we calculate the proportion of non-causal variants that are non-synonymous (see Table 2). Given these settings, the proportions of causal variants among non-synonymous and synonymous variants can be easily derived and are reported for completeness in Table 2.

**Table 2:** Simulation scenarios.

				All		Non-synonymous			
						B_1_		B_2_	
NS:S	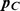	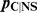	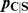	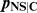	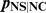	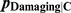	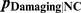	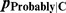	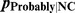
0.6	0.1	0.21	0.03	0.8	0.33	0.8	0.3	0.8	0.1
1.0	0.1	0.16	0.04	0.8	0.47	0.8	0.3	0.8	0.1
1.4	0.1	0.14	0.05	0.8	0.56	0.8	0.3	0.8	0.1
0.6	0.2	0.42	0.06	0.8	0.27	0.8	0.3	0.8	0.1
1.0	0.2	0.32	0.08	0.8	0.42	0.8	0.3	0.8	0.1
1.4	0.2	0.28	0.10	0.8	0.53	0.8	0.3	0.8	0.1

NS:S is the ratio of non-synonymous to synonymous variants; 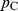 is the percentage of causal variants among the rare variants in a region, with 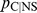 being the percentage of causal variants among the non-synonymous ones, and 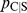 being the proportion of causal variants among the synonymous ones; 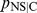 is the proportion of non-synonymous variants among the causal ones; 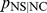 is the proportion of non-synonymous variants among the non-causal ones (these values are calculated based on the NS:S ratio, 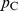, 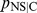). For non-synonymous variants only, we simulate two additional bioinformatic predictors (B_1_ and B_2_), meant to resemble the ‘damaging’ (including possibly and probably) and ‘probably damaging’ annotations from PolyPhen-2. 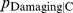 is the proportion of causal variants that are labeled as ‘damaging’ and 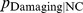 is the proportion of non-causal variants that are labeled as ‘damaging’. Similar notations for ‘probably damaging.’

Furthermore, additional variant annotation tools for non-synonymous variants exist, and are able, for example, to predict the damaging effect of an amino acid substitution (PolyPhen-2 and SIFT), and to assess the extent of evolutionary conservation at a position (GERP++). Therefore, for non-synonymous variants we simulate two additional predictors, as follows. The first bioinformatic predictor (B_1_) for non-synonymous variants is defined as a binary indicator whether a variant is predicted to be damaging or not. Following the empirical results in Cooper et al. [18] we assume that 30% of non-synonymous, non-causal variants are damaging (possibly or probably), and that 80% of non-synonymous, causal variants are damaging (Table 2). A second bioinformatic predictor (B_2_) is also defined as a binary indicator whether a variant is predicted to be probably damaging or not. Again, as in Cooper et al. [18] we assume that 10% of non-synonymous, non-causal variants are probably damaging, and that 80% of non-synonymous, causal variants are probably damaging (Table 2). To assess the effect of using a non-informative predictor, we also simulate a binary predictor with 50% non-synonymous causal and 50% non-synonymous non-causal variants having a value of 1 for this non-informative predictor.

The main goal of the proposed methods is to combine sequencing data with functional predictions about the deleteriousness of variants to identify a set of promising variants, enriched in causal variants. Furthermore, the selected variants can be ranked according to their return counts from the backward elimination procedure, or the estimated 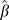 effects from the hierarchical model (ranking based on Z scores gave similar results). We use several measures to assess the performance of the methods. The main measures are: (1) the overall ranking of the true causal variants among the variants in the gene, and (2) the bias and coverage accuracy in the estimation of effect sizes for the variants from the hierarchical model.

### Ranking of causal variants: Simulation results

#### Nonparametric mixture modeling of return counts

In what follows, we apply the backward elimination procedure described in Methodsseparately to non-synonymous and synonymous variants. Since the non-synonymous variants tend to be enriched in causal variants, the sample distribution of return counts obtained for the non-synonymous variants tends to exhibit two separate subgroups (a “non-interesting” group, enriched in null variants, and an “interesting” group, enriched in causal variants). We identify these two groups using an expectation-maximization (EM) algorithm for nonparametric estimation of mixture models and declare as “interesting” those variants belonging to the right tail component (which corresponds to the larger return counts). This is illustrated in Figure S2 which depicts for a simulated example the histogram of return counts for non-synonymous variants with the fitted two-component mixture overlaid.

For synonymous variants, since only a small proportion of causal variants are expected to be synonymous, the sample distribution of return counts often fails to exhibit separate groups in our simulations; therefore we select the variants with the top 20% return counts as “interesting” to be further investigated; although we expect less than 20% of synonymous variants to be causal (indeed in our simulations only 3%–10% of the synonymous variants were causal), we conservatively choose the threshold of 20% to increase the probability of selecting causal variants, especially since for synonymous variants, unlike the non-synonymous ones, we do not make use of additional information in prioritizing variants.

### Ranking of causal variants Non-synonymous vs. synonymous

We employ the backward elimination procedure as well as fit a hierarchical model including the full set of variants and assuming a single functional predictor in the second stage, namely whether a variant is non-synonymous or synonymous. Due to the expected difference in enrichment of causal variants among non-synonymous versus synonymous variants we evaluate the overall ranking of the causal variants separately among non-synonymous and synonymous variants. More explicitly, among the non-synonymous variants selected as “interesting” by the backward elimination procedure we rank the causal variants based on their return counts (this approach is denoted as BE in the figures below). Furthermore, we also use the 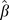 estimates obtained from the hierarchical model to rank the causal variants among all non-synonymous variants (HM) as well as among the non-synonymous variants selected as “interesting” by the backward elimination procedure (HM_S_). For each simulation we take the median of the ranks of the causal variants involved and then compute the median of these estimates across simulations. The ranking for the synonymous variants is done similarly.

**Figure 1:**
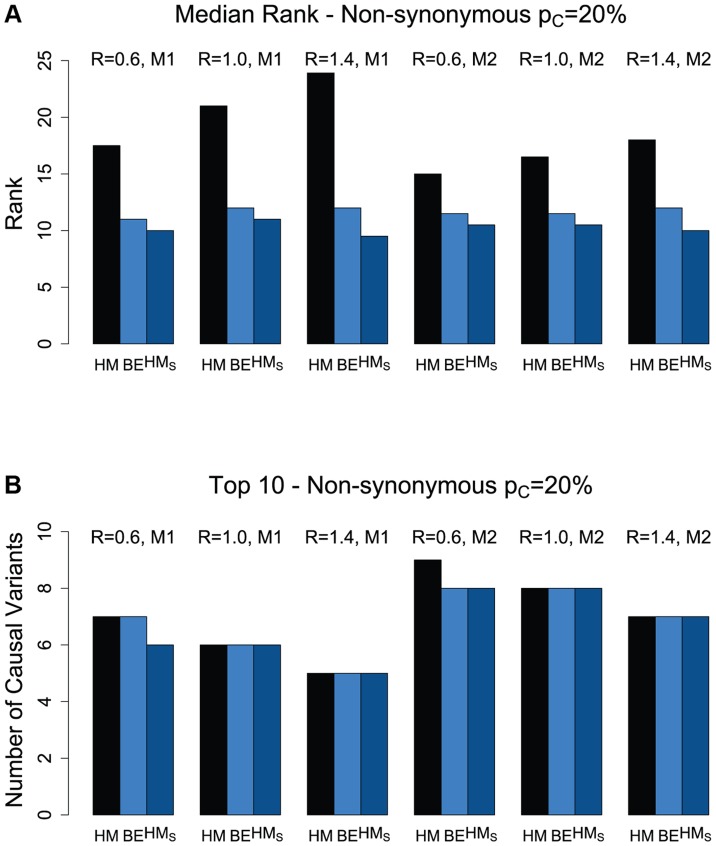
(a) Median rank of causal variants among the non-synonymous variants for two disease models (M1 and M2) and three values for the NS:S ratio (R = {0.6, 1.0, 1.4}). The proportion of causal variants in the region is 20%. HM refers to the original hierarchical model with ranking of the causal variants among the non-synonymous variants, based on their estimated effects; BE refers to the backward elimination procedure for non-synonymous variants; and HM_S_ refers to the ranking of causal variants only among those non-synonymous variants selected by the backward elimination procedure, with ranks based on the estimated effects from the hierarchical model. (b) The number of causal variants in Top 10 for non-synonymous variants.

Figures 1(a) and S3(a) present the median ranks of the causal non-synonymous variants based on the different ranking procedures (HM, BE, and HM_S_). The hierarchical model (titled “HM” in the figure) results in higher median rank (worse performance) than that of the backward elimination procedure (titled “BE” in the figure). This is of course expected due to the smaller number of variants that the backward elimination procedure returns as “interesting” (Figures 1(a) and S3(a)). However, despite excluding a substantial proportion of variants in the backward elimination process (the “non-interesting” category), we show that the top ranked causal variants in the hierarchical model are kept in the selected list (Figures 1(b) and S3(b)). For the scenarios investigated, the number of causal, non-synonymous variants in the top 10 ranked variants varies between 5 and 8 for the case when the percentage of causal variants in a region is 20% (Figure 1(b)), and 3–6 for the case with only 10% causal variants (Figure S3(b)). When looking only among the “interesting” variants from the backward elimination procedure, the overall ranking of causal variants based on the hierarchical model estimates 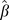 (titled “HM_S_”) is similar to the one based on return counts in the backward elimination procedure (Figures 1 and S3). Therefore the backward elimination method can be used as an effective tool to select and rank a set of promising variants, and reduce the overall list of variants to a smaller, more manageable list, followed by further characterization of these variants’ effects within the framework of the hierarchical model. For all methods, and regardless of disease model, the performance tends to decrease as the non-synonymous to synonymous ratio increases from 0.6 to 1.4 (as the effect of purifying selection becomes weaker).

When looking at the synonymous variants separately, the results are qualitatively similar to the non-synonymous case. However, because only 20% of the causal variants are assumed synonymous, the overall ranking of the few causal variants among the synonymous variants is noticeably worse compared with non-synonymous variants, as expected (Figures S4 and S5). For example, in a region with 20% causal variants, overall we detect between 2 and 3 causal synonymous variants among the top 10 ranked variants, and only 1–2 in a region with 10% causal variants. Due to these high false-discovery rates for synonymous variants, it may be more effective to focus initial efforts for causal variant identification among the functional (non-synonymous and LoF) variants. As genomic annotations become richer for synonymous variants, we expect the discovery of causal variants among synonymous variants to become more accurate.

### Multiple bioinformatic predictors for non-synonymous variants

We evaluate the effect on ranking the causal variants among non-synonymous variants when additional bioinformatic predictors are added to the hierarchical model (in addition to the indicator whether the variant is non-synonymous vs. synonymous). Note that synonymous variants were assigned a bioinformatic predictor of 0.

**Figure 2:**
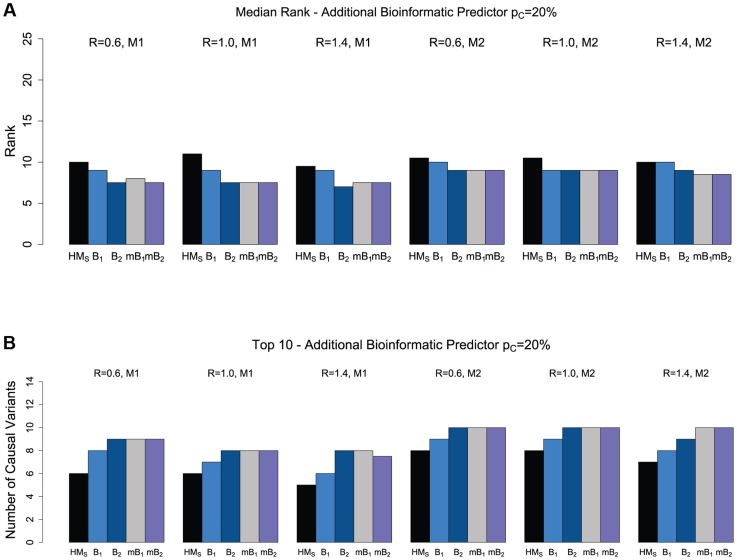
The effect of multiple bioinformatic predictors for non-synonymous variants. Ranking is done only within the set of variants selected by the backward elimination procedure. (a) Median rank of causal variants for two disease models (M1 and M2) and three values for the NS:S ratio (R = {0.6, 1.0, 1.4}). The proportion of causal variants in the region is 20%. HM_S_ refers to the hierarchical model with ranking of the causal variants among the selected non-synonymous variants, based on their estimated effects, B_1_ refers to the hierarchical model with one bioinformatic predictor (B_1_, Table 2), B_2_ refers to the hierarchical model with one bioinformatic predictor (B_2_), mB_1_ refers to the hierarchical model with three bioinformatic predictors (B_1_, B_1_, and B_2_), and mB_2_ refers to the hierarchical model with four bioinformatic predictors (four B_1_s). (b) The number of causal variants in Top 10 for non-synonymous variants.

We restrict attention to ranking only among the “interesting” variants, as selected by the backward elimination procedure. As shown in Figure 2, when we add one bioinformatic predictor (B_1_ or B_2_; see Table 2), the ranking of causal variants improves significantly compared to the original hierarchical model that only uses a binary predictor (whether a variant is non-synonymous or not). The improvement is more pronounced with predictor B_2_, due to the higher specificity of this predictor. For example, for model M1, a non-synonymous to synonymous ratio of 1.4 and 20% causal variants in a region, the median number of causal variants among the top 10 ranked non-synonymous variants increases from 5 (in the original hierarchical model) to 8 when using bioinformatic predictor B_2_. Since we do not always know which of several available bioinformatic predictors may have higher accuracy, the hierarchical model allows us to combine multiple bioinformatic predictors. When combining three bioinformatic predictors (two predictors with the same sensitivity and specificity as B_1_ and one predictor B_2_, all independent), we find that the ranking of causal variants is now similar or superior to the ranking obtained when using only the better of the two bioinformatic predictors (i.e. B_2_). Similarly, when using a combination of four bioinformatic predictors (four predictors with the same sensitivity and specificity as B_1_), the ranking of causal variants is better than using just a single predictor B_1_, and similar to using the more accurate predictor B_2_. These results suggest that using multiple bioinformatic predictors with different accuracies (even multiple weak predictors) can help detection of the causal variants. Similar results are obtained when the proportion of causal variants in a region is 10% (Figure S6).

We have also evaluated the effect of including a non-informative predictor in the analysis, although in practice we expect that functional annotations are correlated with the causal status of a variant. The results are reported in Figures S11 and S12. As shown, including a random (non-informative) bioinformatic predictor does lead to worse performance compared to when such a predictor is not included, although combining an informative predictor (B_1_) together with a non-informative one does help improve the performance. Again, the ability of the hierarchical model to incorporate multiple functional predictors of varying accuracy is an important feature when the best predictors are not known a priori.

It is possible to incorporate one bioinformatic predictor, such as B_1_ or B_2_, in the backward elimination procedure directly (as a weight in the Burden test statistic). We found that for the case of only one bioinformatic predictor, the backward elimination procedure performed similarly with (or slightly worse than) the hierarchical model (Figures S7). However, in general, it is not clear how to choose one single functional annotation from several annotations available. Therefore, the hierarchical model has the important advantage that multiple bioinformatic predictors can be included, and, as shown above, the ranking of the causal variants improves with the addition of several predictors of varying accuracy.

## Effect size estimation for variants in the hierarchical model

As already mentioned, the hierarchical model has distinct advantages when multiple functional predictions are available for variants. In particular, it is possible to provide effect size estimates and standard errors for individual variants, that take into account such diverse functional predictions. As seen in Table S1, for disease model M1 (Table 1), absolute biases of the log odds ratio estimates from the hierarchical modeling approach are similar among the different scenarios while coverages are close to the nominal level of 95%. In comparison, bias is further increased and coverages are under the nominal level of 95% for disease model M2 (which assumes higher odds ratios than model M1, Table 1), though there is a trend towards reduced bias and improved coverage with the addition of stronger bioinformatic predictor(s).

The biases observed here are due to several causes. One main source of bias is the shrinkage phenomenon that occurs with hierarchical models: in this setting of sparse data the model relies heavily on the higher level covariates and as a result the estimated risks of the non-causal variants with high bioinformatic predictor scores will be biased upwards, while the risks of the causal variants with low bioinformatic predictor values will be shrunk down, resulting in increased bias and loss of power, respectively. As the frequency of carriers increases, the model overrides the misclassifications of the higher level covariate, yielding less biased estimates (data not shown). This shrinkage is even more pronounced for model M2, which assumes higher odds ratios for the causal variants (compared to M1), resulting in the poorer performance noted with model M2. An additional source of bias comes from our analyses being conditional on the groupwise (gene-based) test being significant.

## Application to the Dallas Heart Study and *ANGPTL4*

We first show an application of the proposed methods to a well studied re-sequencing dataset for *ANGPTL4* for 3,551 individuals of varied ethnicity from the Dallas Heart Study. Rare and low-frequency variants in this gene have been previously associated with low serum triglyceride levels [35]. We consider log-transformed triglyceride level as our phenotype, and adjust for gender and ethnicity. As in the original study [35], we dichotomize the phenotype by considering the individuals in the lowest quartile as cases, and the individuals in the highest quartile as controls, for a total of 898 individuals with variation in this gene. We identify 20 functional variants (missense, nonsense, and frameshift).

In Table 3 we report the functional variants, ranked by the estimated effects 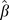 from the hierarchical model taking into account their PolyPhen-2 and GERP_RS scores. Also reported is the return count from the backward elimination procedure (due to the small number of variants in this gene we do not fit the nonparametric mixture model in this example; instead we simply rank all variants). All the top ranked variants that appear only in cases (i.e. the lowest quartile) have been shown by Romeo et al. [36] to severely compromise the function of the protein. In particular, the top ranked variant, p.Lys217Ter, is a nonsense variant that appears only once in an affected individual, and is assumed to interfere with protein synthesis. The second, fourth, seventh and eighth variants have been shown using functional studies to lead to impaired protein secretion. The fifth variant showed reduced ability to inhibit LPL (lipoprotein lipase) activity in vitro, while the sixth variant introduced a premature termination codon [36]. The third variant in the list, p.Glu40Lys, is a missense variant (classified as probably damaging by PolyPhen-2 and as evolutionarily conserved site by GERP_RS), with a frequency of 1.3% in this dataset, and has been shown to be significantly associated with plasma triglyceride levels [35]. However, due to its presence even among controls (i.e. the highest quartile), this variant was not investigated in the functional studies in Romeo et al. [36].

**Table 3:** The 20 functional (non-synonymous, nonsense and frameshift) variants in *ANGPTL4*, with PolyPhen-2 and GERP_RS scores included in the hierarchical model.

variant	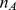	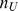	RC	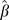	stderr	PolyPhen-2	GERP_RS	SnpEff_effect
p.Lys217Ter	1	0	0.67	2.37	1.63	1	−4.37	non-synonymous (nonsense)
p.Gly361Ser	1	0	0.70	1.54	1.07	0.998	0.741	non-synonymous
p.Glu40Lys	18	5	0.96	1.18	0.43	0.91	4.95	non-synonymous
p.Gly223Arg	1	0	0.69	1.03	0.92	1	4.02	non-synonymous
p.Gly77Arg	1	0	0.75	0.85	0.86	0.694	2.61	non-synonymous
p.Ser302fs	1	0	0.66	0.82	0.93	1	5.37	frameshift
p.Trp349Cys	1	0	0.66	0.82	0.93	1	5.37	non-synonymous
p.Arg384Trp	1	0	0.64	0.73	0.95	1	5.37	non-synonymous
p.Arg278Gln	58	31	0.98	0.71	0.23	0.008	−1.85	non-synonymous
p.Arg336Cys	2	2	0	0.53	0.72	0.971	3.81	non-synonymous
p.Lys245fs	0	1	0.37	0.21	0.92	1	5.37	frameshift
p.Arg371Gln	0	1	0.61	0.21	0.92	1	5.37	non-synonymous
p.Pro251Thr	0	1	0.48	0.20	0.90	0.955	5.08	non-synonymous
p.Glu167Lys	1	0	0.74	−0.08	0.93	0	3.01	non-synonymous
p.Val308Met	0	1	0.42	−0.25	0.86	0.179	1.65	non-synonymous
p.Met41Ile	7	9	0	−0.31	0.49	0.008	4.95	non-synonymous
p.Ser67Arg	0	1	0.50	−0.32	0.91	0.001	0.624	non-synonymous
p.Glu190Gln	5	11	0.57	−0.38	0.45	0.462	2.32	non-synonymous
p.Met1Thr	0	1	0.31	−0.67	0.93	0.013	3	non-synonymous
p.Pro5Leu	0	1	0.47	−0.70	0.94	0.11	4.04	non-synonymous

The variants are sorted according to the log odds ratio estimates (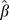) from the hierarchical model. 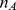 (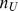) is the minor allele count in cases (controls); RC is the return count from the backward elimination procedure; log odds ratios and their standard errors are estimated from the hierarchical model; PolyPhen-2 score, GERP_RS score, and SnpEff [69] predicted effects are also reported.

We next show an application to a gene with a larger number of functional variants, and for which not much is known on the likely causal variants. Hence the next application is a more difficult example for the proposed methods.

## Application to the Cohen syndrome and *VPS13B*

The Vacuolar Protein Sorting 13 homolog B (*VPS13B*, also known as *COH1*, MIM #607817) is a gene associated with Cohen syndrome (CS, OMIM #216550), a rare autosomal recessive neurodevelopmental disorder overrepresented in Finland and common in Amish, Irish travelers and Greek/Mediterranean founder populations [37], [38]. At least 200 affected individuals of diverse ethnic background have been reported so far with diverse *VPS13B* mutations, including nonsense, missense, splicing, indels, microdeletions and microduplications [38]. Despite clinical heterogeneity in part related to ethnic background, the disorder has core features, including non-progressive intellectual disability, motor clumsiness, postnatal microcephaly, a typical facial gestalt, hypotonia, intermittent neutropenia, and chorioretinal dystrophy [39]. Behavioral disturbances are common among CS individuals, and autistic traits have been reported in cases of greek/mediterranean descent [40]. Furthermore, *VPS13B* mutations have been found in individuals with autism [41] and non-syndromic intellectual disability [42]. It is worth noting that mutations in another member of the *VPS13* gene family (*VPS13A* or *CHAC*, MIM #605978, encoding for a protein known as Chorein), cause chorea-acanthocytosis [43] (MIM #200150), a recessive disorder of acanthocytosis and adult-onset choreic involuntary movements with significant co-morbidity with psychiatric illness [44].

*VPS13B* is also an intolerant gene with a Residual Variation Intolerance Score [45] of −2.44 (top 0.55% most intolerant genes) in Europeans and a similar score for African Americans. We applied the proposed methods to the 166 *VPS13B* variants identified in a whole-exome sequencing autism spectrum disorders (ASD) case/control dataset (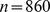; more details on this dataset can be found in Text S1). We tested for association with functional (non-synonymous, nonsense and splice-sites) rare variants in this gene and the Burden test p value was 0.01. We then used the backward elimination algorithm to identify a set of “interesting” (i.e. potentially causal) variants, and for each of these variants we report effect size estimates and standard errors from the hierarchical model. Note that the ratio of non-synonymous to synonymous variants in this gene is 0.84, hence towards the lower end of values in our simulated scenarios.

**Figure 3:**
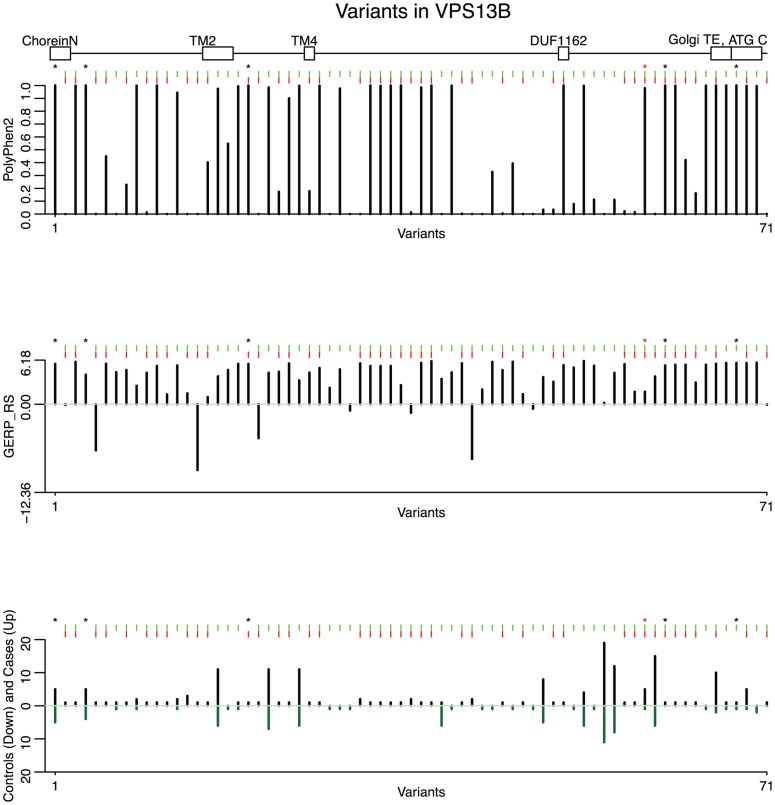
Predicted deleteriousness scores are shown for 71 rare functional variants (non-synonymous, nonsense and splice-sites). From the top, the first plot depicts the PolyPhen-2 score for each variant, the second depicts the GERP_RS score, and the third depicts variant counts for cases (up) and controls (down). Green tick marks indicate a variant contained in an exon, and red ticks indicate that a variant is selected by the backward elimination procedure. LoF variants are marked by a black asterisk; the homozygous probably damaging variant is marked by a red asterisk. The location of five protein domains (ChoreinN, TM2, TM4, DUF1162, Golgi targeting element, and ATG C) are depicted by boxes at the top of the plot (see Figure S10 for a complete view of VPS13B protein domains). Variants are plotted equidistantly on the x-axis.

**Figure 4:**
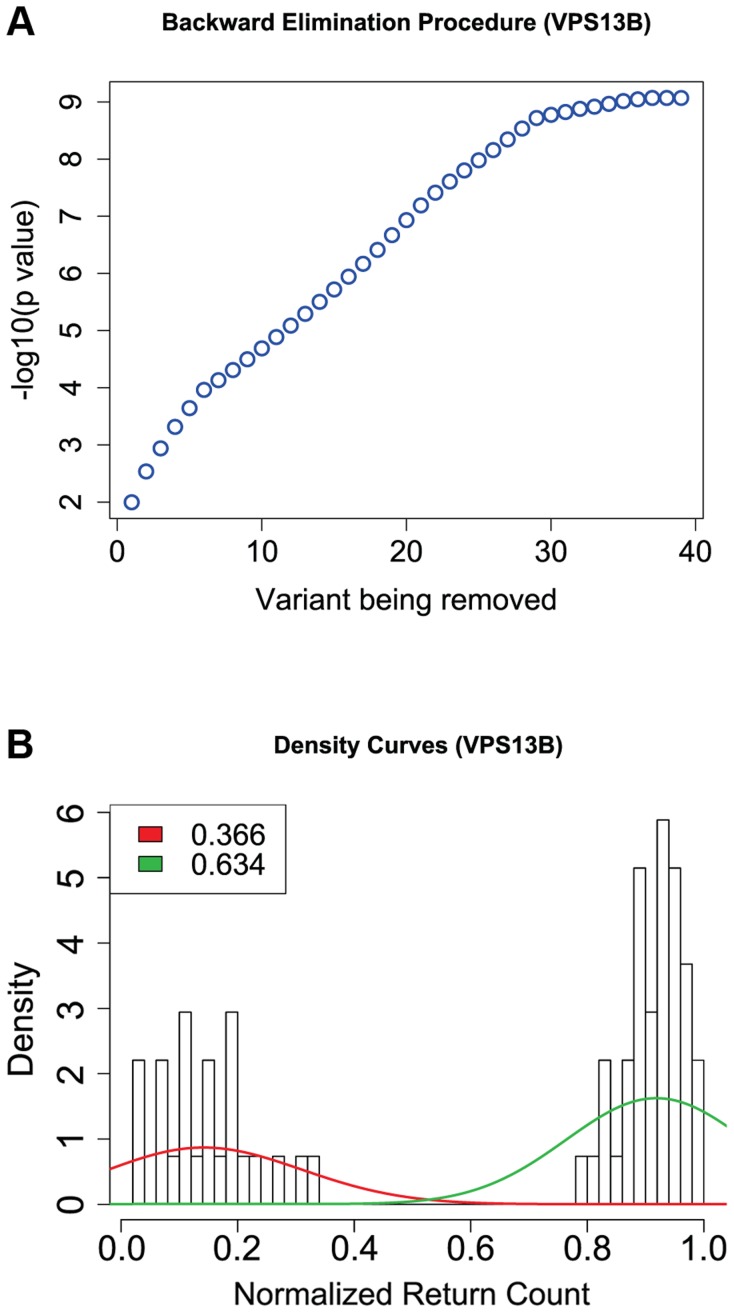
Results from the backward elimination procedure for non-synonymous and splice site variants in *VPS13B*. (a) The change in p value is shown as variants are being removed one by one (when the backward elimination procedure is run once on all non-synonymous variants). (b) Distribution of return counts for non-synonymous and splice site variants in *VPS13B*; overlaid is a fitted mixture with two components.

Of the 166 variants in this gene, we focus on 74 that are non-synonymous, nonsense or splice-sites (two variants affecting the invariant splice acceptor site of the intron between exons 51 and 52 have been excluded from further analyses because they did not validate by Sanger sequencing). Of these, the backward elimination procedure selects 42: 2 of them are LoF (one nonsense and one variant affecting an essential splice site), and of the missense PolyPhen-2 predicts that 14 are probably damaging, 1 possibly damaging, and 25 benign (Figure 3). In Figure 4(a) we show the drop in p value each time a variant is being removed in step 3 of the backward elimination procedure; the process stops when the p value starts to increase as one tries to remove any of the remaining variants. Also shown in Figure 4(b) is the distribution of return counts (from the re-sampling procedure), and overlaid is the fitted mixture with two distinct components. The 42 selected variants belong to the second component of the fitted mixture (these are the “interesting” variants). As a comparison, applying the backward elimination algorithm to the remaining 90 synonymous variants results in no distinguishable “interesting” component (and markedly smaller average return counts compared to the non-synonymous case; Figure S8).

In Table 4 we report the top 20 variants among the selected functional variants (ranked by the estimated effects 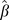 from the hierarchical model), along with their PolyPhen-2 and GERP_RS scores. Noticeably, among the top ranked variants there is a probably damaging variant (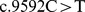, p.Arg3198Trp, annotated on NM_152564.4 and Q7Z768-2, respectively) with 5 variant copies in cases and 1 in controls, with one case being homozygous at the position. Furthermore, the top two variants in the list have both very high C-scores [46] (36 and 35, top 0.1%), based on the recently introduced measure of deleteriousness Combined Annotation-Dependent Depletion (CADD) that integrates diverse genome annotations. The two LoF variants (one nonsense (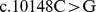, p.Ser3383Ter) and one splice site (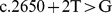)) have been seen only once in cases (i.e. singletons). Notably, the splice variant 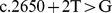 affects the splice donor site of the intron between exons 18 and 19, and a homozygous mutation in the splice acceptor site of the same intron has been identified in an individual suffering from CS [47]. As a first step toward the characterization of the variants, we used Sanger sequencing to validate two cases with the 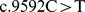 variant and the cases with 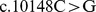 and 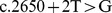 and study their inheritance pattern. This analysis is of particular relevance for singletons, considering that the false discovery rate among those can be high. All variants were validated and found to be inherited (Figure S9). In one family with the 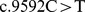, both affected children are homozygous and inherit the variant from their parents (father homozygous and mother heterozygous - Figure S9A). In a second family with 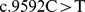, the variant is transmitted from heterozygous parents to one affected child, and untransmitted to the unaffected child (Figure S9B). The 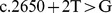 variant is inherited from the mother (Figure S9C), and the 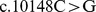 variant is paternally transmitted to both affected children (Figure S9D).

**Table 4:** Top 20 functional (non-synonymous, nonsense and splice site) variants in *VPS13B* (among those selected by the backward elimination procedure), with PolyPhen-2 and GERP_RS scores included in the hierarchical model.

chr	pos	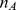	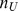	hom_A	hom_U	RC	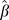	stderr	PolyPhen-2	GERP_RS	SnpEff_effect
8	100874030	10	2	0	0	0.94	0.69	0.41	1	5.66	non-synonymous coding
8	100874154	5	1	0	0	0.96	0.57	0.45	0.997	5.78	non-synonymous coding
8	100861110	15	6	0	0	0.95	0.49	0.36	0.001	3.89	non-synonymous coding
8	100155382	3	0	0	0	1	0.48	0.48	0	1.52	non-synonymous coding
8	100844858	5	1	1	0	0.97	0.48	0.44	0.979	1.75	non-synonymous coding
8	100589771	1	0	0	0	0.92	0.46	0.52	1	2.67	non-synonymous coding
8	100520037	2	0	0	0	1	0.46	0.49	.	5.73	non-synonymous coding
8	100493833	1	0	0	0	0.96	0.45	0.51	0.999	5.08	non-synonymous coding
8	100147957	1	0	0	0	0.85	0.45	0.51	1	5.36	non-synonymous coding
8	100523531	1	0	0	0	0.82	0.45	0.51	0.998	5.36	non-synonymous coding
8	100523389	1	0	0	0	0.96	0.45	0.51	1	5.36	non-synonymous coding
8	100865765	1	0	0	0	0.92	0.44	0.51	1	5.43	stop gained
8	100654700	1	0	0	0	0.89	0.44	0.51	1	6.03	non-synonymous coding
8	100115279	1	0	0	0	0.83	0.44	0.51	0.999	5.94	non-synonymous coding
8	100523656	1	0	0	0	0.89	0.44	0.5	1	5.36	non-synonymous coding
8	100454779	1	0	0	0	0.87	0.44	0.5	0.901	5.71	non-synonymous coding
8	100865927	1	0	0	0	0.89	0.44	0.5	0.998	5.53	non-synonymous coding
8	100712122	2	0	0	0	0.92	0.44	0.59	0	−7.68	non-synonymous coding
8	100286562	1	0	0	0	0.92	0.44	0.51	1	5.65	splice site donor
8	100866155	1	0	0	0	0.93	0.44	0.5	0.42	5.54	non-synonymous coding

The variants are sorted according to the log odds ratios estimates (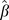) from the hierarchical model. 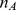 (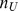) is the minor allele count in cases (controls); hom_A (hom_U) is the number of homozygous genotypes in cases (controls); RC is the return count from the backward elimination procedure; log odds ratios and their standard errors are estimated from the hierarchical model; PolyPhen-2 score, GERP_RS score, and SnpEff [69] predicted effects are also reported.

To understand the impact of the variants on the molecular functions of *VPS13B*, all 42 variants deemed “interesting” by the backward elimination procedure were projected on the protein topology, reconstructed with the Pfam domains (N-terminal region of Chorein, DUF1162, ATG C-terminal domain), the experimentally ascertained Golgi targeting domain [47], and 11 transmembrane domains predicted with TMPred [48] (Figure S10). Benign variants appear scattered along the protein topology, while some of the predicted damaging variants map to known domains, including a missense in the DUF1162 domain and two missense in the Golgi targeting domain. Prediction of the structural changes that can result from the variants using MutPred [49] further revealed two top deleterious missense variants (p.Tyr1428His, predicted to cause gain of disorder (p = 0.006), loss of beta-sheet (p = 0.008) and gain of alpha-helix (p = 0.049); and p.Asp1475Gly, predicted to cause gain of alpha-helix (p = 0.049)). The LoF variants are upstream the Golgi domain, thus they are likely to cause premature insertion of a stop codon, activating nonsense-mediated mRNA decay or producing protein isoforms lacking the Golgi targeting domain. Although the pathological mechanisms caused by *VPS13B* insufficiency or mutations are still unknown, fibroblasts isolated from individuals with CS show severe fragmentation of the Golgi apparatus into ministacks [47], a defect observed in neurodegenerative disorders [50] and hypothesized to precede neuronal cell death [51]. Therefore, the LoF variants might prevent proper localization of VPS13B and disruption of its molecular functions on Golgi assembly or maintenance, triggering the pathological cascades underlying Cohen syndrome and/or autism.

Most of the variants selected are singletons (34 out of 42). As previously mentioned, for singletons accurate bioinformatic predictors about their likely functional effects are essential in order to identify such variants as promising, and hierarchical modeling is a natural framework to incorporate such information. Naturally the false discovery rate among these singletons can be high, and dependent on the sensitivity and specificity of the bioinformatic predictors used to characterize the variants in the hierarchical model. For comparison, in Table S2 we show the top 20 variants among the functional variants selected by the backward elimination procedure with ranking based on return count. No functional prediction score was used in this analysis. Although the more common variants still occur among the top variants in this analysis, for singletons, the hierarchical model gives higher priority to variants with high scores for both PolyPhen-2 and GERP_RS (Table 4). This ability of the hierarchical model to prioritize low frequency variants by taking into account multiple functional predictions is a distinct advantage over ranking based on return count alone (with no consideration of the PolyPhen-2 and GERP_RS scores for the variants).

## Discussion

Pinpointing the rare causal variants among a large number of variants that occur in a genetic region is a difficult challenge, but crucial for follow-up functional studies, and for a better understanding of the molecular mechanisms that lead to disease. For many causal variants that occur only a few times in a dataset, incorporation of external information characterizing the variants (such as bioinformatic predictions on the deleteriousness of a variant) is essential to help prioritize these rare variants. We have described here two complementary statistical methods, that are able to integrate diverse functional annotations on individual variants in a region, and produce a selected list of candidates for causal variants, ranked according to their estimated effect sizes. The backward elimination procedure offers a natural way to select a set of promising variants, while using multiple functional predictors in the hierarchical modeling approach provides more in depth characterization of variants’ effects on disease, and can help boost the power to identify causal variants. We have focused attention here on some of the commonly used annotations for coding regions; however we acknowledge that there are other possible functional genomics annotations available both for the coding and non-coding regions [28] and with continued efforts to improve these functional predictions this list will further expand.

We illustrate the proposed methods through an application to a gene implicated in Cohen syndrome and autism, *VPS13B*. For this gene, we show that among the top selected variants are two LoF variants, and one rare, probably damaging variant that is homozygous in one affected individual. Autosomal recessive mutations associated with autism have been recognized for decades [52]. Recently, whole-exome sequencing has provided strong evidence that rare, recessive LoF variation is a major contributor to risk [53]. It is likely that many recessive missense variants contribute to ASD as well, although there has been insufficient power in whole-exome studies carried out to date to fully explore such variation. *VPS13B* is indispensable for the Golgi apparatus, and genes important for Golgi morphology and function have been linked to autism disorders, including *RAB39B*, mutated in a X-linked intellectual disability associated with autism, epilepsy and macrocephaly [54], and *UBE3A*, responsible of Angelman syndrome [55]. In addition, disturbances in pathways linked to Golgi, e.g. autophagy [56] and protein glycosylation [57], have been associated with autism etiology. Our findings extend the mutational landscape of *VPS13B* in Cohen syndrome and autism and further strengthen the connection between Golgi homeostasis and autism.

A rather large number of selected variants in the backward elimination procedure are singletons. Causal variants that appear as singletons in a dataset are difficult to distinguish from random genetic variation, and accurate functional predictions on such variants are crucial and will help in identifying those singletons more likely to be causal. Currently, it is not uncommon for predictions on the deleteriousness of a variant to be discordant (e.g. predictions from PolyPhen-2 and SIFT), and combining such multiple predictors can be difficult, although aggregate deleteriousness scores, such as Condel [58] and C-score [46], are available. Since the hierarchical model can easily incorporate multiple functional predictions, it has a distinct advantage over methods that cannot consider multiple predictions at once. Indeed, most of the existing groupwise association tests (including the Burden and SKAT tests discussed here) can only use one functional score at a time, and therefore it is not clear how multiple scores (that are sometimes discordant) can be taken into account. It is also worth noting that in the case of existing Burden (or SKAT) tests, a variant with a low functional score (e.g. PolyPhen-2 score close to 0) will be excluded from analysis regardless of the evidence of association that the data suggests (frequency in cases vs. controls). In contrast, in the presence of sufficient case-control frequencies, the hierarchical model places more weight on the larger case-control frequencies overriding the information from the bioinformatic predictors when that information does not support the likelihood of an increased risk. Therefore, the hierarchical model has an advantage also over simple methods to prioritize based on one functional score (e.g. PolyPhen-2) alone.

The use of next-generation sequencing technologies may lead to higher error rates compared to a traditional Sanger sequencing platform. Sequencing errors may be disproportionately present among singletons or very rare variants, especially for larger sample sizes, although for a single gene the number of errors is expected to be relatively small. Therefore, as a first step toward the characterization of the top ranked variants, Sanger sequencing can be used to validate the variants.

Classical variable selection methods (such as ridge regression [59] and LASSO [60]) are natural tools to employ in this setting in which the causal variants are expected to be just a small subset of all sequenced variants. Such methods have recently been applied to sequencing data [61], [62]. However, because these methods have not been developed to handle such sparse data, they have difficulty in selecting very rare variants (such as singletons). Furthermore, it is not clear how one can take into account multiple functional predictions for variants. Further work in this area needs to be done to assess the ability of these classical variable selection approaches to identify rare causal variants. Other existing methods one could use for causal variant prioritization fall into two extremes. Some methods use only data on observed case-control frequencies. For example, in KBAC [11], variants are weighted using data-adaptive weights, reflecting the estimated effect of a variant on the phenotype, and these weights can be potentially used to rank variants. However, as explained above, for rare and low frequency variants it is essential to make use of rich functional genomics annotations. At the other extreme, one can rank variants based on a functional score alone. This latter class of methods has the important drawback, especially in the case of complex traits, that it ignores case-control frequencies, and relies heavily on the accuracy of the bioinformatics predictor. Based on simulation studies, we have shown that hierarchical modeling that takes into account both association evidence coming from the sequencing data, and available functional genomics predictions, have better performance compared with ranking based on a single bioinformatic predictor alone (Text S3 and Figure S13).

We have focused here on the selection of variants that increase risk to disease. However, one can in principle use the proposed methods to identify protective variants. Instead of a Burden type statistic, one can use a SKAT statistic in the backward elimination procedure (also implemented in our software). Similarly, for the hierarchical model, variants with low functional score and higher frequency in controls compared with cases will be candidates for protective variants.

Further improvements to the backward elimination procedure are possible. For example, instead of partitioning variants into two groups based on a binary bioinformatic predictor, such as non-synonymous and synonymous, an alternative would be to calculate stratified false discovery rates [63] or possibly covariate-modulated local false discovery rates [64]. The advantage of such an approach would be that more than one covariate can be added to the backward-elimination procedure, although this point requires further work. Furthermore, information on the location of a variant within a gene or region of interest, e.g. what functional domain it affects, can be important, especially for missense variants. We have previously described scan statistic approaches to identify clusters of rare disease associated variants, and have shown applications to both autism and schizophrenia studies [65], [66], suggesting that incorporating such location information into the backward elimination procedure could improve the identification of causal variants.

The estimates of a rare variant's effect on disease from the hierarchical model can have substantial bias. This happens because in any particular gene or region only a small proportion of variants are expected to be disease causing, and most of the variants represent random genetic variation. Therefore, when estimating odds ratios of causal variants, there is a strong shrinkage effect toward the overall estimate. Incorporation of accurate functional predictors in the hierarchical model is one possible way to help attenuate this bias; further work is needed on finding better ways to reduce the bias.

In addition to their ability to pinpoint likely causal variants, the proposed methods can be used to prioritize variants for genotyping in independent datasets for the purpose of replication or validation. This is relevant when re-sequencing the gene or region in additional datasets is too expensive, and one chooses instead to genotype variants discovered in the original study [67]. Moreover, the proposed approaches can be used at a genome-wide scale, by first selecting the promising genes based on the overall gene-based test or other criteria (e.g. good biological candidate) followed by the backward elimination and hierarchical model approach to prioritize the variants within the genes identified as promising. Such a genome-wide analysis can, for example, identify classes of functional elements or domains enriched among the top variants in the selected genes.

The proposed methods are applicable to case-control or population-based designs. However, family-based designs represent a natural way to identify causal variants. For example, in multiplex families, significant sharing of a non-synonymous mutation among multiple affected relatives can be an important indication of causality. Bayesian approaches in this context have been developed before [68], and further work in this area is worth pursuing.

In summary, we have proposed and investigated two complementary statistical methods to identify causal variants among the naturally occurring genetic variation at a locus. They are able to incorporate sequencing data with various functional predictors on variants, and select a small number of variants that are enriched in causal variants. In the current study, we applied the proposed methods to a gene known to contain risk variants for ASD as proof-of-principle, and identify several interesting variants, including two LoF variants and a homozygous probably damaging variant likely important to autism risk.

## Supporting Information

S1 Figure The effect of removing causal vs. non-causal variants on the p value for the reduced set (Step 2 of the backward elimination procedure). Results are shown for one (typical) simulated dataset with 2000 individuals, in a 25 kb region with 10% causal variants, under model M2. Removing the non-causal variant corresponding to the red circle leads to the largest drop in p value for the reduced set, and hence this variant is being removed in this iteration from the current set (Step 3 of the backward elimination procedure).
(EPS)

S2 Figure Non-parametric mixture fit to sample distribution of return counts in the backward elimination procedure (simulated example). The return counts are for non-synonymous variants, and two groups can be distinguished, one corresponding to the “non-interesting” class, and the other to the “interesting” class.
(EPS)

S3 Figure (a) Median rank of causal variants among the non-synonymous variants for two disease models (M1 and M2) and three values for the NS:S ratio (R = {0.6, 1.0, 1.4}). The proportion of causal variants in the region is 10%. HM refers to the original hierarchical model with ranking of the causal variants among the non-synonymous variants, based on their estimated effects, BE refers to the backward elimination procedure for non-synonymous variants, and HM_S_ refers to the ranking of causal variants only among those non-synonymous variants selected by the backward elimination procedure, with ranks based on the estimated effects from the hierarchical model. (b) The number of causal variants in Top 10 for non-synonymous variants.
(EPS)

S4 Figure (a) Median rank of causal variants among the synonymous variants for two disease models (M1 and M2) and three values for the NS:S ratio (R = {0.6, 1.0, 1.4}). The proportion of causal variants in the region is 20%. HM refers to the original hierarchical model with ranking of the causal variants among the synonymous variants, based on their estimated effects, BE refers to the backward elimination procedure for synonymous variants, and HM_S_ refers to the ranking of causal variants only among those synonymous variants selected by the backward elimination procedure, with ranks based on the estimated effects from the hierarchical model. (b) The number of causal variants in Top 10 for synonymous variants.
(EPS)

S5 Figure (a) Median rank of causal variants among the synonymous variants for two disease models (M1 and M2) and three values for the NS:S ratio (R = {0.6, 1.0, 1.4}). The proportion of causal variants in the region is 10%. HM refers to the original hierarchical model with ranking of the causal variants among the synonymous variants, based on their estimated effects, BE refers to the backward elimination procedure for synonymous variants, and HM_S_ refers to the ranking of causal variants only among those synonymous variants selected by the backward elimination procedure, with ranks based on the estimated effects from the hierarchical model. (b) The number of causal variants in Top 10 for synonymous variants.
(EPS)

S6 Figure The effect of multiple bioinformatic predictors for non-synonymous variants. Ranking is done only within the set of variants selected by the backward elimination procedure. (a) Median rank of causal variants for two disease models (M1 and M2) and three values for the NS:S ratio (R = {0.6, 1.0, 1.4}). The proportion of causal variants in the region is 10%. HM_S_ refers to the hierarchical model with ranking of the causal variants among the selected non-synonymous variants, based on their estimated effects, B_1_ refers to the hierarchical model with one bioinformatic predictor (B_1_, Table 2), B_2_ refers to the hierarchical model with one bioinformatic predictor (B_2_), mB_1_ refers to the hierarchical model with three bioinformatic predictors (B_1_, B_1_, and B_2_), and mB_2_ refers to the hierarchical model with four bioinformatic predictors (four B_1_). (b) The number of causal variants in Top 10 for non-synonymous variants.
(EPS)

S7 Figure The effect of incorporating bioinformatic predictors (B_1_ and B_2_) for non-synonymous variants in the backward elimination procedure (BE_1_ and BE_2_), and in the hierarchical model (B_1_ and B_2_) on the number of causal variants in Top 10. Ranking is done only within the set of variants selected by the backward elimination procedure. Results for two disease models (M1 and M2) and three values for the NS:S ratio (R = {0.6, 1.0, 1.4}) are shown. mB refers to the hierarchical model with three bioinformatic predictors (B_1_, B_1_, and B_2_). (a) 20% causal. (b) 10% causal.
(EPS)

S8 Figure Distribution of return counts from the backward elimination procedure applied to synonymous variants in *VPS13B*; overlaid is a fitted mixture with two components.
(EPS)

S9 Figure Molecular validation and transmission pattern analysis for two cases with the c.9592C>T (p.Arg3198Trp) variant (A, B), the c.2650+2T>G singleton LoF (C) and the c.10148C>G (p.Ser3383Ter) singleton missense (D). For each family, the electropherogram of the relevant region is shown below the corresponding individual.
(EPS)

S10 Figure Schematic representation of VPS13B (Q7Z768-2) in the Golgi membrane and the variants identified in this study. The Chorein N-terminal domain is shown in magenta, DUF1162 in cyan, and ATG C-terminal domain in green. The Golgi targeting domain overlaps with the ATG C and is shown in purple. Amino acids affected by LoF are shown in black, probably damaging missense in red, possibly damaging in yellow, and benign in grey. The arrow points to the homozygous probably damaging variant (p.Arg3198Trp).
(EPS)

S11 Figure The effect of incorporating a random bioinformatic predictor R for non-synonymous variants in the hierarchical model (20% causal). Ranking is done only within the set of variants selected by the backward elimination procedure. Results for two disease models (M1 and M2) and three values for the NS:S ratio (R = {0.6, 1.0, 1.4}) are shown. RB_1_ refers to the hierarchical model with two bioinformatic predictors (a random one, i.e. R, and B_1_). (a) Median rank of causal variants among the non-synonymous variants. (b) The number of causal variants in Top 10 for non-synonymous variants.
(EPS)

S12 Figure The effect of incorporating a random bioinformatic predictor R for non-synonymous variants in the hierarchical model (10% causal). Ranking is done only within the set of variants selected by the backward elimination procedure. Results for two disease models (M1 and M2) and three values for the NS:S ratio (R = {0.6, 1.0, 1.4}) are shown. RB_1_ refers to the hierarchical model with two bioinformatic predictors (a random one, i.e. R, and B_1_). (a) Median rank of causal variants among the non-synonymous variants. (b) The number of causal variants in Top 10 for non-synonymous variants.
(EPS)

S13 FigureROC curves of the z-values estimated from a hierarchical model including PolyPhen-2 scores and an indicator for the non-synonymous vs. synonymous status as the higher level covariates (solid curves), and ROC curves based on ranking variants using the PolyPhen-2 scores alone (dashed curves); 10% of variants are assumed truly causal variants; associations between the PolyPhen-2 scores and the causal status vary from odds ratio of 2 (blue) to 4 (red); 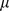, the effect size as a function of standard deviations, is assumed to be 0.5; estimates are averaged across 400 simulations (see Text S3 for more details on the simulation setup).(EPS)

S1 Table Absolute biases and coverage probabilities when estimating variant effects in the hierarchical model, for the simulation scenarios in Table 2. Results for two disease models (M1 and M2 - Table 1) are shown. Several functional predictors are used in the hierarchical model: non-synonymous vs. synonymous (NS vs. S), B_1_, B_2_, and a scenario with three functional, independent predictors: two B_1_'s and one B_2_.
(PDF)

S2 TableTop 20 functional (non-synonymous, nonsense and splice-site) variants in *VPS13B*; only a functional vs. synonymous indicator is used as a binary functional predictor. The variants are sorted according to return count. 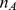 (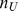) is the minor allele count in cases (controls); hom_A (hom_U) is the number of homozygous genotypes in cases (controls); RC is the return count from the backward elimination procedure; log odds ratios and their standard errors are estimated from the hierarchical model. SnpEff predicted effects are also reported. PolyPhen-2 and GERP_RS scores are also reported but they are not used in the backward elimination procedure.(PDF)

S1 Text Describes details about the Autism Sequencing Dataset.
(PDF)

S2 Text Describes details about the nonparametric estimation of multivariate mixtures.
(PDF)

S3 Text Presents comparisons between variant ranking from hierarchical model and variant ranking from PolyPhen-2 scores.
(PDF)
