# Supplementary material for: Identification of Rare Causal Variants in Sequence-Based Studies: Methods and Applications to VPS13B, a Gene Involved in Cohen Syndrome and Autism
Source: PLoS Genet. 2014 Dec 11;10(12):e1004729. doi: 10.1371/journal.pgen.1004729 (PMC4263785; doi:10.1371/journal.pgen.1004729)
Supplement: S1 Table — Absolute biases and coverage probabilities when estimating variant effects in the hierarchical model, for the simulation scenarios in Table 2. Results for two disease models (M1 and M2 - Table 1) are shown. Several functional predictors are used in the hierarchical model: non-synonymous vs. synonymous (NS vs. S), B1, B2, and a scenario with three functional, independent predictors: two B1's and one B2. (PDF) [file pgen.1004729.s014.pdf]

			NS vs. S		B_1		B_2		2 B_1 and B_2	
NS:S	p_C	Model	Bias	Coverage	Bias	Coverage	Bias	Coverage	Bias	Coverage
0.6	0.1	M1	0.17	0.95	0.17	0.96	0.15	0.97	0.17	0.97
		M2	0.28	0.90	0.26	0.91	0.22	0.93	0.23	0.94
1.0	0.1	M1	0.17	0.95	0.18	0.96	0.16	0.97	0.19	0.97
		M2	0.29	0.90	0.27	0.90	0.24	0.92	0.25	0.94
1.4	0.1	M1	0.18	0.95	0.18	0.96	0.16	0.97	0.19	0.97
		M2	0.29	0.90	0.28	0.90	0.24	0.92	0.26	0.93
0.6	0.2	M1	0.20	0.94	0.19	0.95	0.18	0.95	0.19	0.96
		M2	0.37	0.87	0.33	0.88	0.29	0.90	0.29	0.92
1.0	0.2	M1	0.22	0.93	0.20	0.94	0.18	0.95	0.20	0.96
		M2	0.40	0.87	0.36	0.87	0.31	0.90	0.31	0.91
1.4	0.2	M1	0.22	0.93	0.21	0.94	0.18	0.95	0.20	0.96
		M2	0.41	0.87	0.37	0.87	0.32	0.89	0.32	0.91
