# Supplementary material for: Identification of Rare Causal Variants in Sequence-Based Studies: Methods and Applications to VPS13B, a Gene Involved in Cohen Syndrome and Autism
Source: PLoS Genet. 2014 Dec 11;10(12):e1004729. doi: 10.1371/journal.pgen.1004729 (PMC4263785; doi:10.1371/journal.pgen.1004729)
Supplement: S2 Table — Top 20 functional (non-synonymous, nonsense and splice-site) variants in VPS13B; only a functional vs. synonymous indicator is used as a binary functional predictor. The variants are sorted according to return count. () is the minor allele count in cases (controls); hom_A (hom_U) is the number of homozygous genotypes in cases (controls); RC is the return count from the backward elimination procedure; log odds ratios and their standard errors are estimated from the hierarchical model. SnpEff predicted effects are also reported. PolyPhen-2 and GERP_RS scores are also reported but they are not used in the backward elimination procedure. (PDF) [file pgen.1004729.s015.pdf]

chr	pos	n_A	n_U	hom_A	hom_U	RC	$\hat{\beta}$	stderr	PolyPhen-2	GERP_RS	SnpEff_effect
8	100133585	1	0	0	0	1	0.36	0.32	0.085	4.79	non-synonymous
8	100155382	3	0	0	0	1	0.42	0.31	0.001	1.52	non-synonymous
8	100520037	2	0	0	0	1	0.39	0.32	0.483	5.73	non-synonymous
8	100205126	1	0	0	0	0.97	0.36	0.32	0.001	-9.22	non-synonymous
8	100844858	5	1	1	0	0.97	0.40	0.31	0.409	1.75	non-synonymous
8	100493833	1	0	0	0	0.96	0.36	0.32	0.997	5.08	non-synonymous
8	100874154	5	1	0	0	0.96	0.43	0.30	0.998	5.78	non-synonymous
8	100523389	1	0	0	0	0.96	0.36	0.32	0.997	5.36	non-synonymous
8	100654621	1	0	0	0	0.96	0.36	0.32	0.693	5.79	non-synonymous
8	100861110	15	6	0	0	0.95	0.43	0.27	0.003	3.89	non-synonymous
8	100654424	2	0	0	0	0.94	0.38	0.31	0.006	-1.2	non-synonymous
8	100874030	10	2	0	0	0.94	0.50	0.29	0.999	5.66	non-synonymous
8	100155285	1	0	0	0	0.94	0.36	0.32	0.003	1.37	non-synonymous
8	100796685	1	0	0	0	0.94	0.36	0.32	0.999	5.47	non-synonymous
8	100844849	1	0	0	0	0.94	0.36	0.32	0.003	1.75	non-synonymous
8	100287477	1	0	0	0	0.94	0.36	0.32	0.003	-4.76	non-synonymous
8	100866155	1	0	0	0	0.93	0.37	0.32	0.918	5.54	non-synonymous
8	100128062	1	0	0	0	0.93	0.37	0.32	0.005	-6.46	non-synonymous
8	100286562	1	0	0	0	0.92	0.36	0.32	0.99	5.65	splice site donor
8	100865765	1	0	0	0	0.92	0.36	0.32	0.99	5.43	stop gained
