# Supplementary material for: Identification of Rare Causal Variants in Sequence-Based Studies: Methods and Applications to VPS13B, a Gene Involved in Cohen Syndrome and Autism
Source: PLoS Genet. 2014 Dec 11;10(12):e1004729. doi: 10.1371/journal.pgen.1004729 (PMC4263785; doi:10.1371/journal.pgen.1004729)
Supplement: S1 Text — Describes details about the Autism Sequencing Dataset. (PDF) [file pgen.1004729.s016.pdf]

Autism Spectrum Disorder Sequencing data

The ASD case-control dataset has been sequenced as part of the ARRA Autism Sequencing Collaboration (AASC), and is publicly available through dbGaP (dbGaP Study Accession: phs000298.v1.p1). The dataset consists of 488 ASD cases and 372 controls of European ancestry, and whole-exome sequencing was performed at the Broad Institute using an Illumina HiSeq2000 platform. Data was processed with Picard[1] and BWA[2] to map reads to hg19. Variants were called using the Genome Analysis Toolkit[3] and only those variants that passed standard quality control filters were analyzed.

References

1. DePristo MA, Banks E, Poplin R, Garimella KV, Maguire JR, Hartl C, Philippakis AA, del Angel G, Rivas MA, Hanna M et al. (2011) A framework for variation discovery and genotyping using next-generation DNA sequencing data. *Nat Genet* 43: 491–498.

2. Li H, Durbin R (2010) Fast and accurate long-read alignment with Burrows-Wheeler transform. *Bioinformatics* 26: 589–595.

3. McKenna A, Hanna M, Banks E, Sivachenko A, Cibulskis K, Kernytsky A, Garimella K, Altshuler D, Gabriel S, Daly M et al. (2010) The Genome Analysis Toolkit: A MapReduce framework for analyzing next-generation DNA sequencing data. *Genome Res* 20: 1297–1303.
