# Supplementary material for: Identification of Rare Causal Variants in Sequence-Based Studies: Methods and Applications to VPS13B, a Gene Involved in Cohen Syndrome and Autism
Source: PLoS Genet. 2014 Dec 11;10(12):e1004729. doi: 10.1371/journal.pgen.1004729 (PMC4263785; doi:10.1371/journal.pgen.1004729)
Supplement: S2 Text — Describes details about the nonparametric estimation of multivariate mixtures. (PDF) [file pgen.1004729.s017.pdf]

Nonparametric estimation of multivariate mixtures

We present here the EM-like algorithm that performs nonparametric estimation in multivariate mixtures [1]. This algorithm is implemented in an R package (R Development Core Team, 2008) called `mixtools` available online from the Comprehensive R Archive Network (CRAN). Using the same notation as in [1], suppose the vectors $\vec{X}_1, \ldots, \vec{X}_n$ are a simple random sample from a finite mixture of $m > 1$ arbitrary distributions. The density of each $\vec{X}_i$ may be written as

$$g(\vec{x}_i) = \sum_{j=1}^{m} \lambda_j \prod_{k=1}^{r} f_{jk}(x_{ik}),$$

where f_{jk} are univariate density functions and r is the number of coordinates of the $\vec{X}_i$ vector.

Given initial values $\vec{\varphi}^0 = (\vec{\lambda}^0, \vec{f}^0)$, the algorithm iterates between the following three steps until convergence of the $\vec{\lambda}$, that is, we stop the algorithm when the maximum difference between two consecutive values of λ is sufficiently small. Then for iterations $t = 1, 2, \ldots$:

1. E-step: Conditional on the data and $\vec{\varphi}^t$, for all $i = 1, \ldots, n$ and $j = 1, \ldots, m$, calculate the posterior probabilities that subject i belongs to component j

$$p_{ij}^t = P_{\vec{\varphi}^t}(Z_{ij} = 1 | \vec{x}_i),$$

where Z_{ij} are Bernoulli random variables indicating the subject i comes from component j.

2. M-step: Set $\lambda_j^{t+1} = \frac{1}{n} \sum_{i=1}^{n} p_{ij}^t$, for $j = 1, \ldots, m$.

3. Nonparametric (Kernel) density estimation step using a standard normal density

function as the kernel function and with the bandwidth chosen according to the rule of thumb due to [2].

More details and formulas can be found in [1]. Note that this algorithm is not an EM algorithm in the usual sense because there is no likelihood that this algorithm may be shown to maximize, but the authors retain the “EM” name because the algorithm strongly resembles a true EM algorithm for the parametric mixture case.

References

1. Benaglia T, Chauveau D, Hunter DR (2009) An EM-like algorithm for semi- and non-parametric estimation in multivariate mixtures. *Journal of Computational and Graphical Statistics* 18: 505–526.

2. Silverman BW (1986) Density Estimation, London: Chapman and Hall.
