# Supplementary material for: Identification of Rare Causal Variants in Sequence-Based Studies: Methods and Applications to VPS13B, a Gene Involved in Cohen Syndrome and Autism
Source: PLoS Genet. 2014 Dec 11;10(12):e1004729. doi: 10.1371/journal.pgen.1004729 (PMC4263785; doi:10.1371/journal.pgen.1004729)
Supplement: S3 Text — Presents comparisons between variant ranking from hierarchical model and variant ranking from PolyPhen-2 scores. (PDF) [file pgen.1004729.s018.pdf]

ROC analysis comparing the hierarchical model with a PolyPhen-2 based ranking

We investigate the discriminatory ability of our hierarchical modeling approach and that of the ranking based on the PolyPhen-2 scores alone using simulations based on the 166 variants sequenced in the *VPS13B* gene (see Section 3.4). We generated the truly causal variants based on a logistic regression with PolyPhen-2 as the only predictor, assuming an association between the causal status and the PolyPhen-2 score of magnitude 2 or 4 and assuming the proportion of truly causal variants to be 10%. The case-control status was generated following the same data generating mechanism as in [1]. In particular, for carriers of causal variants we generated a continuous phenotype from a normal distribution with mean $\mu = 0.5$ and a standard deviation of 0.2 while for non-carriers we used a standard normal distribution. Cases were defined as the individuals with phenotype values above the median while the remaining individuals were classified as controls. The hierarchical model included the PolyPhen-2 scores and the non-synonymous vs. synonymous indicator as the higher level covariates. Synonymous variants were assigned a PolyPhen-2 score of 0.

The ROC curves for the two different methods are displayed in Figure S13 for the different degrees of associations between the causal status of a variant and the bioinformatic predictor and assuming $\mu = 0.5$. Solid curves correspond to ROC curves based on the Z values estimated from the hierarchical model, while dashed curves correspond to rankings based on the PolyPhen-2 scores. The hierarchical modeling approach is shown to have superior discriminatory ability to the PolyPhen-2 score method, with biggest improvements

observed when the functional score is weakly associated with the causal status (OR=2, blue curves). As the association of the bioinformatic/functional predictor with the causal status gets stronger (OR=4, red curves), the discriminatory ability of the two methods also improves.

References

1. Ladouceur M et al. (2012) The empirical power of rare variant association methods: results from sanger sequencing in 1,998 individuals. *PLoS Genet* 8: e1002496.
